# Comparative effects of lymphatic drainage and soft tissue mobilization on pain threshold, shoulder mobility and quality of life in patients with axillary web syndrome after mastectomy

**DOI:** 10.1186/s12905-023-02762-w

**Published:** 2023-11-10

**Authors:** Tahniyat Amir Meer, Rabiya Noor, Muhammad Salman Bashir, Mehwish Ikram

**Affiliations:** 1 Faculty of Rehabilitation and Allied Health Sciences, Riphah International University, Lahore Campus, Lahore, Pakistan; 2 School of Health Sciences, University of Management and Technology, Lahore, Pakistan

**Keywords:** Axilla, Breast Neoplasm, Manual lymphatic drainage, Muscle strength dynamometer, Muscle stretching exercises

## Abstract

**Purpose:**

The purpose was to compare the effects of manual lymphatic drainage and soft tissue mobilization on pain threshold, shoulder mobility and quality of life in patients with axillary web syndrome.

**Methods:**

This randomized clinical trial was conducted on 36 breast cancer patients with developed axillary web; participants were randomly divided into two groups. One group was treated with manual lymphatic drainage; the other group was treated with soft tissue mobilizations in addition to therapeutic exercises, i.e., stretching, strengthening and range of motion (ROM) exercises. The duration of treatment was four weeks (5 sessions/week), with therapeutic exercises as a common treatment protocol. Outcome measures were Breast-Cancer specific quality of life questionnaires, Disabilities of the Arm, Shoulder and Hand (DASH), Numeric Pain Rating Scale (NPRS), Patient Specific Functional Scale (PSFS), Dynamometer and Goniometer. All outcome measure readings were recorded at baseline and the end (4th week) of the treatment.

**Results:**

The compliance of the variable distribution with normal distribution was verified using the Shaphiro-Wilk test. Parametric tests were applied, and both groups showed significant effects (p < 0.05) in pairwise comparison (paired t-test). The comparison group analysis (independent t-test) showed that there was no significant difference in pain, upper limb strength, range of motions and fatigue component of quality of life questionnaire parameters (p > 0.05). Two parameters (DASH, PSFS) and one component of the quality of life questionnaire (global health) showed a significant difference (p < 0.05).

**Conclusion:**

Manual lymphatic drainage showed more improvement in functional movements. It was concluded that both groups, manual lymphatic drainage and soft tissue mobilization groups were clinically equally effective.

**Trial Registration Number:**

This trial is registered at ClinicalTrial.gov PRS under trial number NCT05463185 on date 18/07/2022.

## Introduction

Axillary web syndrome (AWS) is a condition experienced by many patients post-surgically, mainly after the mastectomy with axillary lymph node dissection (ALND) procedures. There are palpable cords in the axilla, and the surrounding tissues are suffered from pain and restricted joint motion of the affected side [1]. It is a post-surgical complication that is common but underrated, experienced by 6–86% of patients that have undergone axillary dissection surgery, and usually appears as a complication 5–8 weeks post-surgically. However, pain and range of motion restriction due to AWS are reported in 74% of subjects [2, 3]. There are visible and palpable cords, webs or adhesions in the axilla, breast, antecubital space, chest walls, hands and arms post-ALND. It causes shoulder movement difficulty; abduction is mainly restricted due to cording [4, 5].

AWS originates initially at the axilla and moves forward towards the medial arm, then the anteromedial forearm, and in severe cases, it involves the base of the thumb. Studies report that AWS is self-resolving and usually takes three months to resolve automatically, but some exceptions may persist further in some cases. Occurrence of AWS is generally reported within eight weeks post-surgically but can also appear over three months. The risk of developing AWS increases with age, ethnicity, prolonged surgery, low BMI, and complications in the healing process [6].

AWS is often accompanied by restricted movement at the axilla, pain and lymphedema. Physical examination showed that the main characteristic of AWS is cording and webbing in superficial tissues of the axilla, chest, and arm, causing painful and restricted joint movement. The cord can be visible and easily palpable when the arm is fully extended and abducted [7]. Movement such as shoulder flexion and abduction are the most limited, thus restricting patients from moving their limbs due to disabling pain [8].

The effectiveness of physiotherapy in AWS management for upper limb disability, pain reduction and functional limitations is positively highlighted in the literature. It suggests early rehabilitation to avoid the aggravation of symptoms [8–11]. Manual Lymphatic Drainage (MLD) uses specific techniques like a gentle massage, scooping, clearing and flowing. The main techniques used are gentle massage and scooping in MLD [12, 13]. Soft Tissue Mobilization (STM) is also effective in slowing down the webbing process and has high literature support when applied with stretching exercises [11, 13–15]. MLD is a unique therapy provided to reduce lymphedema, improving lymph circulation and enhancing tissue mobility to assist proper and careful lymph drainage; this technique is explicitly based on the knowledge of the lymphatic system to reduce lymphedema [12, 16]. This technique increases the contractility of surrounding soft tissues of the area, increases the elasticity of lymphatic vessels and increases their contractions in natural ways. Thus, stimulating lymph drainage, reducing lymph blockage and assisting fluid from the blocked area towards the open large lymphatic vessels [17]. A skilled myofascial soft tissue mobilization technique (STM) is inspired by a “cross friction message’’ to treat tissue scars and fibrosis, thus reviving muscular and skin contractions and elasticity. The micro-traumas with controlled pressure break adhesion formed within soft tissue by breaking them [18, 19].

Strength training is an essential part of approximately all rehabilitation programs for individuals of all ages to enhance strength [20]. As tissue remodelling capacity improves as a result of strength training, there is a betterment in the healing capacity of tissues, making fast repair and healing from injury possible [21]. Stretching throughout the available range and above initiates changes in the musculoskeletal system’s contractile and non-contractile elements, thus, lowering muscle stiffness and contracture prevention [22, 23]. Prolonged inactivity or disuse can significantly decrease the range of motion affecting joints and tissues. Therefore therapeutic activities are advised in almost all rehabilitation programs to prevent contracture formation and loss of movement [24].

This study’s findings will develop awareness regarding the non-pharmacological management of patients with axillary web syndrome by improving their pain symptoms, shoulder mobility and quality of life. The results of this study can help formulate future guidelines for the management of AWS that help clinicians treat such patients more efficiently. The purpose of this study was to compare the effects of manual lymphatic drainage and soft tissue mobilization on pain threshold, shoulder mobility and quality of life in patients with axillary web syndrome.

## Methodology

The study was a randomized clinical trial. This trial was registered at ClinicalTrial.gov PRS under trial number NCT05463185 on date 18/07/2022. The study was started after approval from the ethical research committee of Riphah International University, Lahore Campus, Pakistan, with the reference number REC/RCR & AHS/22/0511. The data were collected at the Allied Hospital (Oncology ward, Breast Clinic and Physiotherapy ward), Faisalabad. The epitool software calculated the sample size of 36 after adding a 10% attrition rate [12].

### Participant’s inclusion criteria

Breast cancer patients with pain (NPRS > 3) points and four weeks after surgery [12]. The participant’s ages ranged from 18 to 60 years, with visible and palpable cords in the axilla, arm and breast after surgery. Participants with shoulder abduction were limited to the range of 70–80 and shoulder extension to 20–30 degrees. Patients with minor-level lymphedema or grade I lymphedema were included.

Patients with chemotherapy and radiotherapy, acute thrombosis, musculoskeletal disorders, skin problems, infections, osteoarthritis, rheumatoid arthritis, rotator cuff syndrome, adhesive capsulitis, and any post-surgical condition were excluded. A convenience sampling technique has been used.

They were requested to participate in the study via informed consent. Patients were randomly allocated into groups A and B via a lottery method by an unbiased physiotherapist. The outcome assessor and participants were blinded from the group allocation.

#### Group A (MLD)

This group has received manual lymphatic drainage and stretching, strength, and range of motion exercises.

#### Group B (STM)

This group has received soft tissue mobilization and stretching exercises, strength training and range of motion exercises.

### Tools (outcome measures)

#### Quality of life questionnaires

Breast cancer-specific QOL questionnaires European Organization for the Research and Treatment of Cancer Quality of Life Questionnaire (EORTC QLQ-30, EORTC QLQ-BR23). EORTC QLQ-30 has 30 questions covering five different functional scale domains: physical, functional, cognitive, social and emotional (additional three symptomatic scales including pain, fatigue, nausea and vomiting). EORTC BR-23 consists of 23 questions covering both functional and symptomatic scales [25]. Both questionnaires have same scoring patteren each question is provided with four options where 1 is the lowest score (indicating no difficulty at all) while 4 is the highest score indicating very much difficulty in performing certain chores of daily living. Both quality of life questionnaires have tested validity according to literature [12].

#### DASH

The DASH questionnaire assesses functional measures that have been asked with 30 different questions regarding the disabilities of the hand, arm and shoulder and answers are recorded as no difficulty to mild, moderate, severe and unabling levels. Each question have lowest score of 1 which means there is no difficulty in performing certain task and highest score of 5 indicating that person is unable to perform task in question. At least 27 questions must be answered to complete assessment. Scoring is reported between 0 and 100, with indicating higher disability if scores are high. DASH is reported to be having high validity and reliability in functional assessment scales and is used worldwide in many studies [12, 26].

#### NPRS

NPRS is an outcome measure that is a segmented numeric version of a visual analogue scale in which respondents select a whole number (0–10 integers) that best reflects the intensity of a patient’s pain [27]. There are total of 11 points ranging from 0 to 10, where 0 indicates no pain while 10 strong pains. The common format is horizontal bar or line and NPRS is anchored by term using pain and severity extremes. Patients rate their pain according to their experience for last whole week. Have high validity and reliability for pain assessment in cancer patients [12].

#### PSFS

PSFS is an outcome valuable measure for quantifying activity limitations and functional consequences for patients with orthopaedic problems. The scoring is from 0 (unable to perform activity) to 10 (able to perform activity at the same level as before injury). Studies reported its validity and reliability regarding cancer rehabilitations [15, 28].

#### Dynamometer

Dynamometer was used for strength measurement of the involved limb. There were rest periods of 5–10 s between each contraction to avoid muscular fatigue and discomfort. All the measurements and assessments were done in the sitting position. The maximum contraction duration was 5 s to measure proper strength [29].

#### Goniometer

Goniometer was used to measure the ranges of the upper limb and shoulder. All assessments were performed in the sitting position. Each movement was performed thrice, and average values were analyzed accordingly [30].

### Treatment approach

Both groups were examined for their upper limb and shoulder muscles before and after the training sessions, and assessments were done with outcome measures [12, 15]. Group A received manual lymphatic drainage and therapeutic exercises; Group B received soft tissue mobilization and therapeutic exercise. Both groups received 5 sessions per week for four weeks. After 4th week, assessments of both groups were taken [12, 15].

### Group A interventions (manual lymphatic drainage)

Group A received manual lymphatic drainage on the shoulder region and upper limb. Manual lymphatic drainage was provided proximal to distal starting from the axillary region and then moving towards the shoulder, arm, and forearm, making 5–7 strokes at each part. Techniques like gentle massage and scooping were given for 25 min to patients in sitting or lying positions depending upon their comfort [12].

### Group B interventions (soft tissue mobilization)

Group B received soft tissue mobilization of the axillary cord and arm (upper limb) for 20 min. Patient was in lying position either supine and prone depending upon the targeted muscles. Three minutes on each part (axilla, upper arm front and back side, lower arm front and back side) with a round of 1 min and 10 s of stretch (a total of three rounds in one session) [15].

Limb was positioned in elevation with the help of pillows or support during and after treatment sessions. At rest, the limb was adapted to elevation position with the help of pillows to avoid return of lymph.

### Common interventions

Therapeutic interventions, including strengthening exercises, range of motion exercises and stretching exercises, were equally performed by all the patients in both groups. These exercises were performed in the sitting position while the therapist was in standing position and body mechanics were properly maintained to avoid any work-related injury [12, 15]. Rehabilitation protocol exercises included.

1. Strength training: 5 days per week for four weeks, intensity mild to moderate, 5 to 7 repetitions (3 sets followed by resting duration of 30 s between sets) and resistance training with light weights and bands.
2. Stretching: 5 days per week for four weeks, moderate intensity 7–10 repetitions, with a 5-sec hold for each stretch beyond range (resting duration of 5 s in between repetititions), passive and active manual stretches.
3. ROM exercises: 5 days per week for four weeks, moderate intensity, 5–7 repetitions, with a 5-sec hold (followed by resting period of 5 s in between repetitions) within range, passive and active ROM exercises.

Ten minutes of warm-up were done through this rehabilitation protocol. After the specific therapy, a cool-down period of 10 min was done to reduce cramping and fatigue.

### Data Analysis

Statistical analysis was performed using SPSS version 25. Statistical significance was set at P = 0.05. The normality of data was assessed through the Shapiro-Wilk test. Change over time; the difference between pre-treatment and post-treatment readings was calculated using Paired sample t-test as data were parametric. Difference between groups; Independent sample t-test was used. This parametric test was used to compare two populations at different intervals.

## Results

Demographic data was measured using descriptive statistics such as mean and standard deviations. The mean age of Group A (MLD) was 49.84 ± 12.35 years, while that of Group B (STM) was 45.88 ± 11.95 years. The mean weight of both groups, MLD and STM was 71.42 ± 13.483 kg and 75.23 ± 12.0 kg, respectively. The mean body mass index (BMI) of the participants of MLD and STM was 27.06 ± 4.28 kg/m^2^ and 28.82 ± 3.9 kg/m^2^, respectively.

**Fig. 1:**
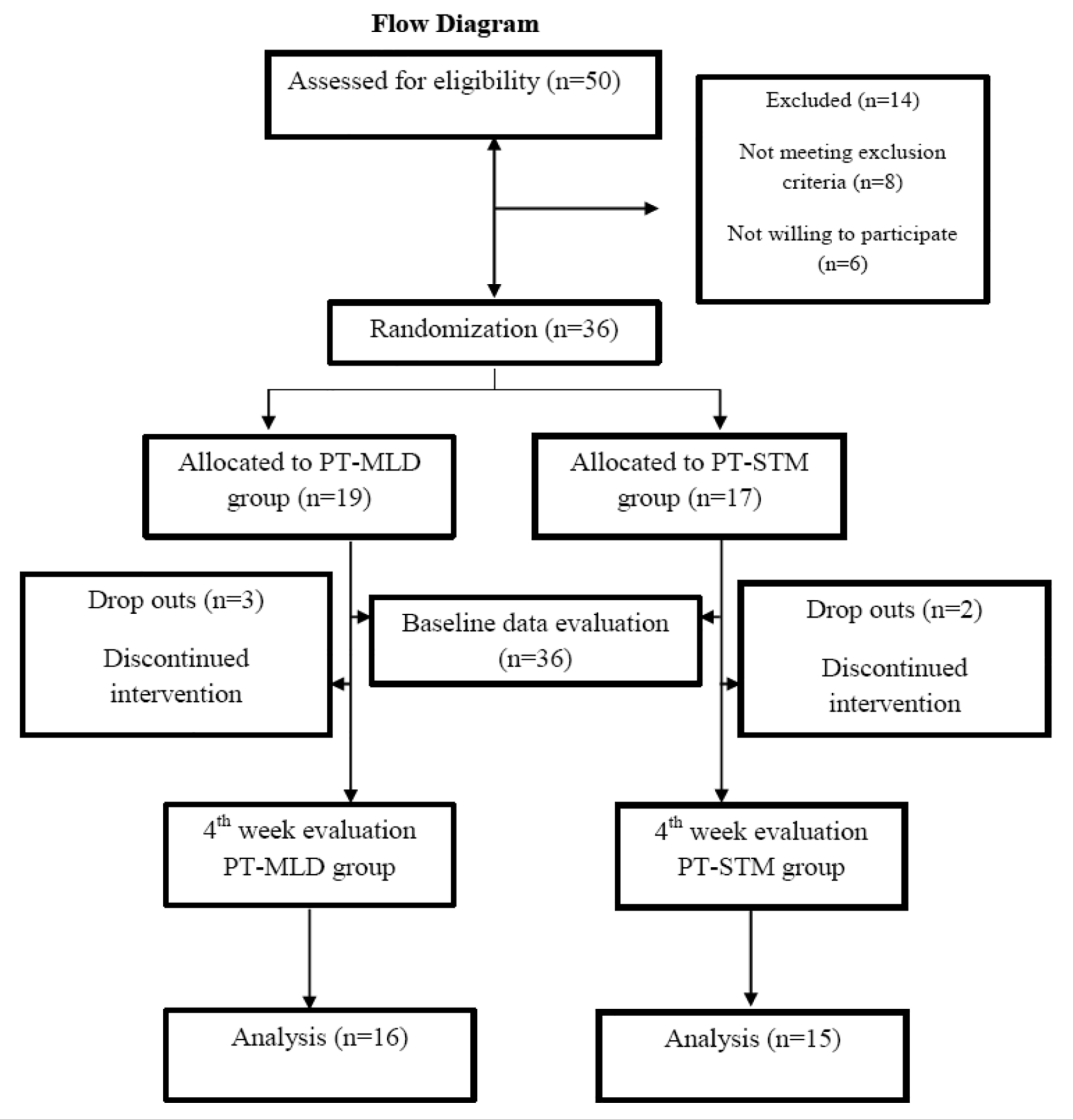
Flow Diagram

The baseline data of both groups at the pre-intervention stage had no significant differences; both groups were homogeneous p > 0.05 (independent t-test). A total of 36 participants were enrolled and 5 patients discontinued treatment due to some personal reasons. Participants recruitment and dropped-out flow chart are shown in Fig. 1.

The Shapiro-Wilk test showed that the data were parametric. The paired sample-t test was used to compare pre-treatment and post-treatment values within the groups. There were significant differences in pre and post-treatment values of all the variables in both groups. (shown in Tables 1, 2, 3 and 4)

**Table 1:** Across and within group analysis of EORCT QLQ C-30

**Global Health**	**Group A**	**Group B**	**Mean difference**	**p-value**
Pre-Value	78.31 ± 6.61	80.22 ± 8.33	-1.91	0.346
Post-Value	30.01 ± 8.32	38.99 ± 9.82	-8.98	0.009
Mean difference	48.3	41.23		
p-value	< 0.05	< 0.05		
**Physical Function**	**Group A**	**Group B**	**Mean difference**	**p-value**
Pre-Value	37.90 ± 11.34	42.93 ± 13.80	-5.03	0.210
Post-Value	70.41 ± 14.99	62.91 ± 10.60	7.5	0.113
Mean difference	-32.51	-19.98		
p-value	< 0.05	< 0.05		
**Role Function**	**Group A**	**Group B**	**Mean difference**	**p-value**
Pre-Value	24.99 ± 12.17	22.03 ± 9.59	2.96	0.579
Post-Value	82.29 ± 14.23	71.87 ± 15.77	10.42	0.059
Mean difference	-57.3	-49.84		
p-value	< 0.05	< 0.05		
**Emotional Function**	**Group A**	**Group B**	**Mean difference**	**p-value**
Pre-Value	15.44 ± 7.79	16.03 ± 9.56	-0.59	0.687
Post-Value	67.76 ± 15.77	56.76 ± 14.66	11	0.050
Mean difference	-52.32	-40.73		
p-value	< 0.05	< 0.05		
**Cognitive Function**	**Group A**	**Group B**	**Mean difference**	**p-value**
Pre-Value	31.10 ± 9.61	32.28 ± 12.12	-1.18	0.550
Post-Value	76.08 ± 8.57	72.95 ± 12.01	3.13	0.403
Mean difference	-44.98	-40.67		
p-value	< 0.05	< 0.05		
**Social Function**	**Group A**	**Group B**	**Mean difference**	**p-value**
Pre-Value	21.87 ± 10.03	25.40 ± 15.23	-3.53	0.477
Post-Value	59.99 ± 19.79	51.08 ± 26.20	8.91	0.288
Mean difference	-38.12	-25.68		
p-value	< 0.05	< 0.05		

**Table 2:** Across and within group analysis of quality of EORTC BR-23

**Fatigue symptom**	**Group A**	**Group B**	**Mean difference**	**p-value**
Pre-Value	53.46 ± 12.97	59.70 ± 18.08	-6.24	0.153
Post-Value	35.06 ± 15.41	41.66 ± 14.34	-6.6	0.220
Mean difference	18.4	18.04		
p-value	< 0.05	< 0.05		
**Pain Symptom**	**Group A**	**Group B**	**Mean difference**	**p-value**
Pre-Value	20.82 ± 7.45	22.21 ± 9.46	-1.39	0.630
Post-Value	71.87 ± 14.53	67.28 ± 14.35	4.59	0.377
Mean difference	-51.05	-45.07		
p-value	< 0.05	< 0.05		
**Arm Symptom**	**Group A**	**Group B**	**Mean difference**	**p-value**
Pre-Value	35.41 ± 16.33	34.74 ± 15.01	0.67	0.796
Post-Value	74.99 ± 8.60	77.07 ± 9.48	-2.08	0.521
Mean difference	-39.58	-42.33		
p-value	< 0.05	< 0.05		
**Breast Symptom**	**Group A**	**Group B**	**Mean difference**	**p-value**
Pre-Value	54.16 ± 12.90	49.47 ± 12.35	4.96	0.204
Post-Value	84.74 ± 7.46	77.97 ± 11.56	6.77	0.058
Mean difference	-30.58	-28.5		
p-value	< 0.05	< 0.05		

**Table 3:** Across and within group analysis of DASH, NPRS, PSFS and MMT

**DASH**	**Group A**	**Group B**	**Mean difference**	**p-value**
Pre-Value	81.48 ± 5.06	80.50 ± 6.08	0.98	0.063
Post-Value	34.67 ± 2.67	56.24 ± 9.25	-21.57	0.000
Mean difference	46.81	24.26		
p-value	< 0.05	< 0.05		
**NPRS**	**Group A**	**Group B**	**Mean difference**	**p-value**
Pre-Value	7.50 ± 0.93	7.46 ± 0.82	0.04	0.782
Post-Value	2.85 ± 1.24	3.54 ± 0.63	-0.69	0.061
Mean difference	4.65	3.92		
p-value	< 0.05	< 0.05		
**PSFS**	**Group A**	**Group B**	**Mean difference**	**p-value**
Pre-Value	5.13 ± 1.08	4.92 ± 1.11	0.21	0.071
Post-Value	8.27 ± 0.72	7.37 ± 0.75	0.9	0.002
Mean difference	-3.14	-2.45		
p-value	< 0.05	< 0.05		
**MMT**	**Group A**	**Group B**	**Mean difference**	**p-value**
Pre-Value	2.25 ± 0.57	2.18 ± 0.65	0.07	0.089
Post-Value	4.43 ± 0.51	4.06 ± 0.57	0.37	0.061
Mean difference	-2.18	-1.88		
p-value	< 0.05	< 0.050		

[Abbreviations: DASH = Disabilities of the Arm, Shoulder and Hand; NPRS = Numeric Pain Rating Scale; MMT = Manual Muscle Testing]

**Table 4:** Across and within group analysis of the ranges of the shoulder

**Flexion**	**Group A**	**Group B**	**Mean difference**	**p-value**
Pre-Value	105 ± 17.1	100 ± 22.35	5	0.779
Post-Value	160 ± 10.32	158 ± 13.60	2	0.777
Mean difference	-55	-58		
p-value	< 0.05	< 0.05		
**Extension**	**Group A**	**Group B**	**Mean difference**	**p-value**
Pre-Value	29.37 ± 7.71	30.93 ± 8.60	-1.56	0.557
Post-Value	48.43 ± 3.01	46.85 ± 7.72	1.58	0.457
Mean difference	-19.06	-15.92		
p-value	< 0.05	< 0.05		
**Abduction**	**Group A**	**Group B**	**Mean difference**	**p-value**
Pre-Value	101.2 ± 25.78	85.62 ± 20.64	15.58	0.173
Post-Value	164.3 ± 7.27	158.1 ± 17.59	6.2	0.199
Mean difference	-63.1	-72.48		
p-value	< 0.05	< 0.05		

An Independent t-test was used to compare changes between MLD and STM groups post-intervention. Results showed that the MLD and STM both groups are equally effective in treating axillary web syndrome. There was no significant difference (p > 0.05) in quality of life outcome measures, NPRS, MMT and range of motions. One component of the quality of life questionnaire (global health), DASH and PSFS outcome measures showed a significant difference (p < 0.05). Manual lymphatic drainage was considered more beneficial based on these two parameters, but both groups showed clinical effectiveness when we see in paired sample t-test (shown in Tables 1, 2, 3 and 4).

## Discussion

The purpose of this study was manual lymphatic drainage versus soft tissue mobilization for managing pain threshold, shoulder mobility and quality of life in patients with axillary web syndrome. A total of 36 participants were included in the study who met the inclusion criteria, out of which 32 completed the study. Baseline exercise therapy was the same in both groups. The mean age and BMI of the participants were 47.34 ± 10.67 and 27.96 ± 3.78, respectively. Both treatment plans were clinically effective; only two outcome measures, DASH and PSFS in the manual lymphatic drainage group showed significant effects. Limb circumference was measured initially and at the end of the intervention period (4th week). There was little change in measurement like 1-2 cm as there was type-1 lymphedema.

Therapeutic exercises (stretching, strengthening and range of motion exercises) were performed in both groups as a baseline treatment therapy. Intensities were adjusted between mild to moderate to avoid complications like fatigue, cramping and soreness. Stretching exercises were incorporated to improve tissue flexibility and mobility, with 7–10 repetitions, with each stretch held for 5 s beyond the available range. According to the literature, the most fundamental benefit of stretching exercises is tissue mobility, as stretching initiates changes in contractile and non-contractile elements of the tissues, thus lowering tissue stiffness and preventing contracture formation [21]. ROM exercises were added to increase the range of the limb by 5–7 times, repeatedly moving the limb in the available range. Literature provides evidence to add ROM exercise protocol to all rehabilitation programs [24, 31].

In previous studies, manual lymphatic drainage was mainly used for axillary web syndrome. Self MLD and physical exercise was also used for the treatment of lymphedema and showed good results [32–34]. In a previous study MLD and active exercise group showed no significant results in the range of motion and wound healing [34].

In comparison to the recent study, the effects of MLD were seen in a study for the treatment of AWS. DASH, NRS, arm volume, quality of life questionnaire, and shoulder flexor strength were measured after a 4-week intervention. NRS and arm volume showed significant results with PT in combination with MLD [12]. A systematic review of conservative treatments of lymphoedemas after mastectomy was conducted by Moseley Al and colleagues. MLD is a unique technique that helps reduce lymphedema, improves lymphatic circulation, and enhances tissue mobility to reduce lymphedema chances [16]. Leduc O and colleagues stated in their study that manual lymphatic drainage increases the elasticity of surrounding tissues and lymphatic vessels and increases the flow more naturally. Stimulates drainage in lymph vessels reduces blockage and moves fluid toward open and healthy lymph nodes and larger lymph vessels [17]. MLD reduces clinical symptoms with the combined use of vacuum sealing and, reduces pain, upper limb disability function and improves the quality of life in AWS patients [35].

Soft tissue mobilization (STM) is also beneficial in treating AWS, as it improves ranges by breaking the cord effectively and is helpful in pain reduction [11, 18]. This technique uses ‘’myofascial release,” thus breaking the web or cord instantly by application of controlled pressure over the area. As a result, cord breaking initiated through micro-traumas activates the healing cascade. This cascade helps by resolving tissue adhesions and traumas, reducing pain and increasing the limb’s range of motion; this technique was followed previously by Fowler and Wilson JK in their study in 2000 [19]. In some studies of physical therapy interventions, different techniques were used in combination to remove the problem. Axillary web syndrome was treated with scar massage, skin traction, myofascial release and soft tissue mobilization [36].

Physiotherapy treatment was considered to be a conservative method for axillary web syndrome. With combined multiple interventions, of PT treatment plan was designed that includes lymphatic drainage, manual therapy, stretches, strengthening and ROM exercises and soft tissue mobilization/massage that proved to be an effective protocol for axillary web syndrome patients [37]. The limitation of the study was the manual lymphatic drainage treatment time was more than soft tissue mobilization. In future, we can study STM with long-duration treatment plans with the addition of follow-ups.

## Conclusion

Global health, Shoulder disability and patient-specific function scale showed more progress in the manual lymphatic drainage group. Both groups were clinically effective in improving the quality of life, pain, strength and shoulder range of motion in patients with axillary web syndrome.

## Data Availability

Data will be available at a reasonable request from the corresponding author.

## Abbreviations

NPRS: Numeric Pain Rating Scale
DASH: Disabilities of the arm, shoulder and hand
MMT: Manual Muscle Testing
PSFS: Patient Specific Functional Scale
ROM: Range of Motion
CONSORT: Consolidated Standards of Reporting Trials
